# Microbiome data: tell me which metrics and I will tell you which communities

**DOI:** 10.1093/ismeco/ycaf125

**Published:** 2025-07-24

**Authors:** Alessandro Fuschi, Alessandra Merlotti, Daniel Remondini

**Affiliations:** Department of Physics and Astronomy, University of Bologna, Bologna, BO 40127, Italy; Department of Physics and Astronomy, University of Bologna, Bologna, BO 40127, Italy; Department of Physics and Astronomy, University of Bologna, Bologna, BO 40127, Italy

**Keywords:** microbiome, microbial ecology, beta-diversity, ecological distance metrics, ordination methods, multivariate analysis, compositional data, next-generation sequencing, Bray–Curtis dissimilarity, Aitchison distance

## Abstract

In microbial community studies, analyzing diversity is crucial for uncovering ecological complexity. However, the intrinsic characteristics of Next-gen sequencing data challenge the use of Euclidean metrics for estimating proximity and correlation. Consequently, a variety of distance measures have been developed within ecological frameworks. In this study, we compare several of these metrics—including Bray–Curtis, Canberra, Jensen–Shannon, Hellinger, Euclidean, and Aitchison distances—demonstrating how the choice of metric can significantly influence the interpretation of microbial community structures. Among these, Aitchison distance specifically defined for compositional data shows markedly different behavior from the others, highlighting different features related to the data. We consider two real-world examples: the human gut microbiome sampled using 16S rRNA sequencing with multiple measurements for different patients (G-HMP2) and urban sewage environmental metagenomes collected over time at different sites through shotgun sequencing (E-WADES). We show that, for the same dataset—independently on the sequencing technique or on the sampling context—the community structure depends strongly on the choice of specific metrics. This can be explained by the mathematical properties of the chosen metrics and the specific characteristics of microbiome data, namely their high heterogeneity in species abundance. This provides clear insights into how distance metrics influence interpretation and assists in choosing the most appropriate one for the study objectives.

## Introduction

Next-generation sequencing (NGS) technologies have revolutionized our ability to study microbial communities across a wide range of ecosystems [1, 2]. These advances have enabled detailed exploration of microbial diversity, revealing the complexity and variability of microbial ecosystems. In ecological research, the study of diversity occupies a central role, considering α, β, and γ diversity indexes [3] to capture the full spectrum of microbial community variability [4]. We narrow our focus to β-diversity [5, 6] which compares the species composition across samples, in contrast to α-diversity that quantifies the diversity within a single sample and γ-diversity that assesses overall diversity across multiple habitats.

Microbial abundance data consist of non-negative values—positive or zero—and are often highly sparse, with zeros arising from either true biological absence or technical limitations like undersampling. In addition, these datasets typically exhibit high skewness and dimensionality [7], making them challenging to analyze with conventional statistical methods such as Euclidean distance, which is commonly used to estimate proximity or correlation but is poorly suited to this data structure [8].

To overcome these limitations, a number of alternative distance measures have been developed, particularly within ecological and probabilistic frameworks. Among them, Bray–Curtis dissimilarity has gained widespread use for its simplicity and effectiveness in comparing species abundances across samples. Other commonly adopted metrics—such as Jensen-Shannon divergence, Canberra, and Hellinger distances—are derived from probability theory and are well suited to abundance profiles that are normalized to sum to one, as these resemble probability distributions in practice.

In contrast, compositional data theory is based on different principles for analyzing relative abundance data, treating them as points in a simplex—the natural space for closed data, where components carry meaning only in relation to each other [9].

We focused on distance metrics that compare vectors of positive abundance values directly, excluding those based on presence/absence (e.g. Jaccard) or incorporating phylogenetic relationships (e.g. weighted UniFrac).

This study explores how different distance metrics affect the interpretation of microbiome data, using Principal Coordinate Analysis (PCoA) to visualize sample relationships and Permutational Multivariate Analysis of Variance (PERMANOVA) to assess the contribution of biological factors to community variability.

We initially explored a range of distance metrics—including Bray–Curtis, Canberra, Jensen–Shannon, and Hellinger—and found that most yielded results broadly similar to Bray–Curtis, with the exception of Aitchison distance, which produced notably different patterns due to its compositional nature (see Supplementary Fig. 1). For this reason, we focus our comparison in the main text on Bray–Curtis dissimilarity, as a widely adopted ecological metric, and Aitchison distance, as the only formal compositional approach, to illustrate how the choice of metric can influence the interpretation of microbiome variation.

For both datasets, distance matrices were computed using Bray–Curtis dissimilarity and Aitchison distance via the *vegdist* function from the *vegan* [10] package. For Aitchison distance, zeros in the relative abundance data were replaced using the Bayesian-multiplicative method implemented in the *cmultRepl* function from the *zCompositions* [11] package. PCoA was performed using the cmdscale function from the **stats** package, and PERMANOVA on distance matrices was conducted with adonis2 (vegan), evaluating the marginal effect of each factor.

**Figure 1:**
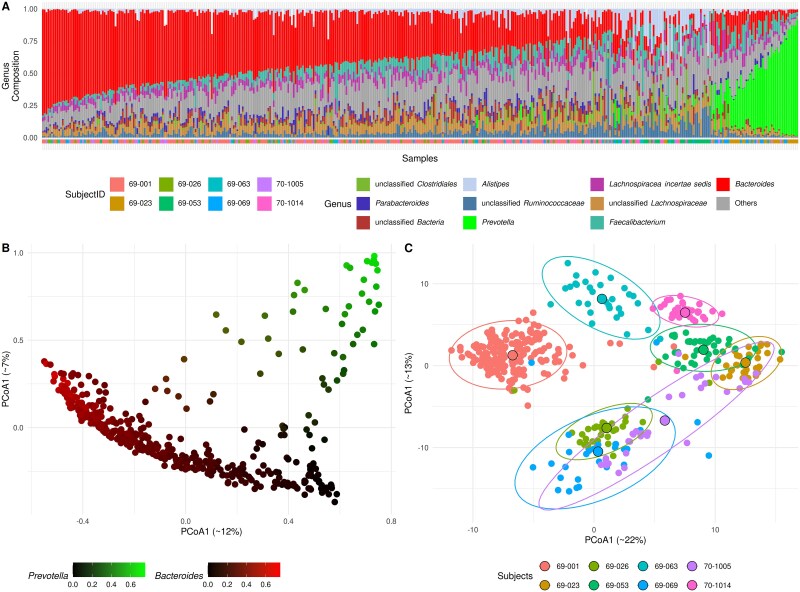
Comparative Analysis of Microbial Community Structures in the G-HMP2 Dataset Using Bray-Curtis and Aitchison Distances: A) Sample composition at genus level arranged according to the abundance of Bacteroides minus Prevotella. B-C) PCoA plot based on Bray-Curtis dissimilarity (left) and on Aitchison distance (right); In panel C, ellipses show subject-wise dispersion as 95% confidence intervals (multivariate Student’s T-distribution), and large black-edged points mark group centroids.

**Figure 2:**
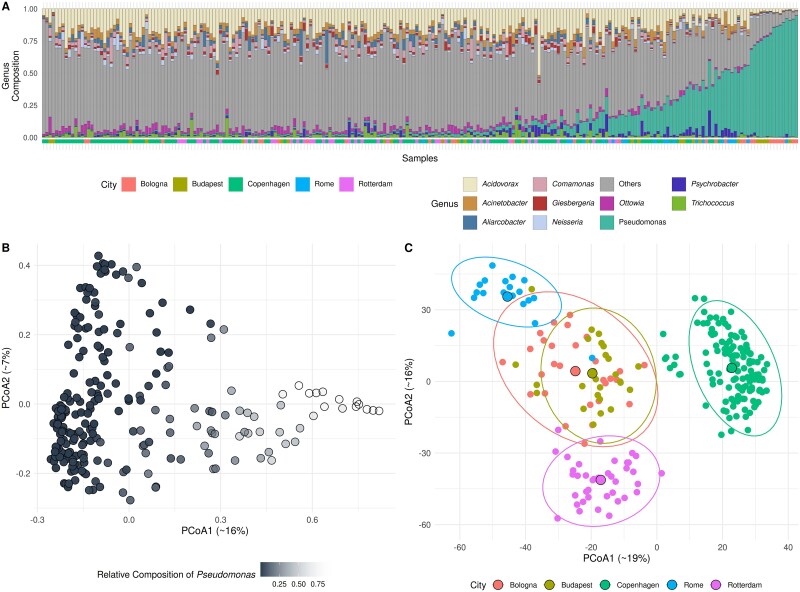
Comparative Analysis of Microbial Community Structures in the E-WADES Dataset Using Bray-Curtis and Aitchison Distances: A) Genus-level composition of the samples, showing the top 10 most abundant genera, sorted by the presence of *Pseudomonas*. B-C) PCoA plot based on Bray-Curtis dissimilarity (left) and on Aitchison distance (right); In panel C, ellipses show city-wise dispersion as 95% confidence intervals (multivariate Student’s T-distribution), and large black-edged points mark group centroids.

Metadata incorporated into the final visualizations included both contextual and microbial factors: in the G-HMP2 dataset, subject identity and the relative abundances of *Prevotella* and *Bacteroides*, which often dominated the profiles (Fig. 1A)—consistent with patterns previously described in human enterotype studies [12]; in the E-WADES dataset, geographical origin and the abundance of *Pseudomonas*, which in some samples became predominant (Fig. 2A) and strongly influenced the microbial community composition.

In the G-HMP2 dataset, Bray-Curtis distance emphasized differences driven by the dominant genera *Bacteroides* and *Prevotella* (Fig. 1B), while Aitchison distance revealed a structure more strongly associated with individual subjects (Fig. 1C). Consistently, PERMANOVA results showed that subjects explained more variance in Aitchison distance (R^2^ = 0.36) than in Bray–Curtis (R^2^ = 0.15), while *Bacteroides/Prevotella* composition had a stronger effect on Bray–Curtis (R^2^ = 0.24 vs. 0.02 for Aitchison).

In the E-WADES dataset, Bray–Curtis distance emphasized the influence of *Pseudomonas* (Fig. 2B), a genus that was highly abundant in certain samples (Fig. 2A), while Aitchison distance more clearly captured differences related to sampling location (Fig. 2C). As for the G-HMP2 dataset, the variance explained by each factor mirrored the visual pattern: city accounted for more variation in Aitchison distance (R^2^ = 0.33 vs. 0.17 for Bray–Curtis), whereas *Pseudomonas* abundance contributed more to Bray-Curtis (R^2^ = 0.13 vs. 0.04 for Aitchison).

To support these findings, we provide additional results in the Supplementary Material: Supplementary Fig. 2 replicates the PCoA analysis using UMAP for dimensionality reduction, further confirming that patient identities in G-HMP2 and sampling locations in E-WADES appear more distinct with Aitchison’s compositional metrics.

As these two cases illustrate, across different biological contexts (human or environmental) and sequencing strategies (16S rRNA gene or whole-genome shotgun), non-compositional metrics—including Bray-Curtis and other abundance-sensitive measures—tend to emphasize differences driven primarily by the most abundant taxa. In contrast, Aitchison distance, grounded in compositional theory, compares taxa through their abundance ratios. Thus, when a single taxon becomes dominant and occupies a large proportion of the sequencing reads, the relative ratios among less abundant taxa remain unaffected, allowing Aitchison distance to preserve and highlight the underlying overall compositional structure of the microbiome. These findings suggest that the choice of distance metric can substantially influence downstream interpretation of microbiome data, particularly when taxa are unevenly distributed—as is often the case. Therefore, selecting an appropriate metric should be guided by the characteristics of the data and the specific biological questions being addressed. In practical terms, Bray–Curtis and similar non-compositional distances are more influenced by dominant taxa when abundance distribution is highly skewed, while Aitchison distance provides a more balanced view by incorporating variation from both dominant and less abundant taxa.

## Supplementary Material

Microbiome_Metrics_Supplementary_ycaf125

## Data Availability

The datasets analyzed and the code used during this study are available in the GitHub repository at https://github.com/Fuschi/Microbiome_Metrics. G-HMP2 raw data included in this study are hosted on the NIH Human Microbiome Project 2 site (https://portal.hmpdacc.org) with no restrictions on its use, and exome sequencing data are available at dbGaP under Study Accession phs001719. v1.p1. Both raw and processed data are also hosted on the Stanford iPOP site (http://med.stanford.edu/ipop.html). For the E-WADES project, sequencing reads, metagenomic assemblies, and MAGs have been deposited in the European Nucleotide Archive (ENA) under accession code PRJEB68319, with databases available for download from the Figshare public data repository (doi:10.6084/m9.figshare.25016147.v1).
