# Supplementary material for: Microbiome data: tell me which metrics and I will tell you which communities
Source: ISME Commun. 2025 Jul 24;5(1):ycaf125. doi: 10.1093/ismeco/ycaf125 (PMC12342790; doi:10.1093/ismeco/ycaf125)
Supplement: Microbiome_Metrics_Supplementary_ycaf125 [file microbiome_metrics_supplementary_ycaf125.pdf]

Microbiome data: tell me which metrics and I will tell you which communities - Supplementary material

Alessandro Fuschi[1], Alessandra Merlotti[1], Daniel Remondini[1,*]

*Corresponding author

1. Department of Physics and Astronomy, University of Bologna, Bologna 40127, IT.

* daniel.remondini@unibo.it

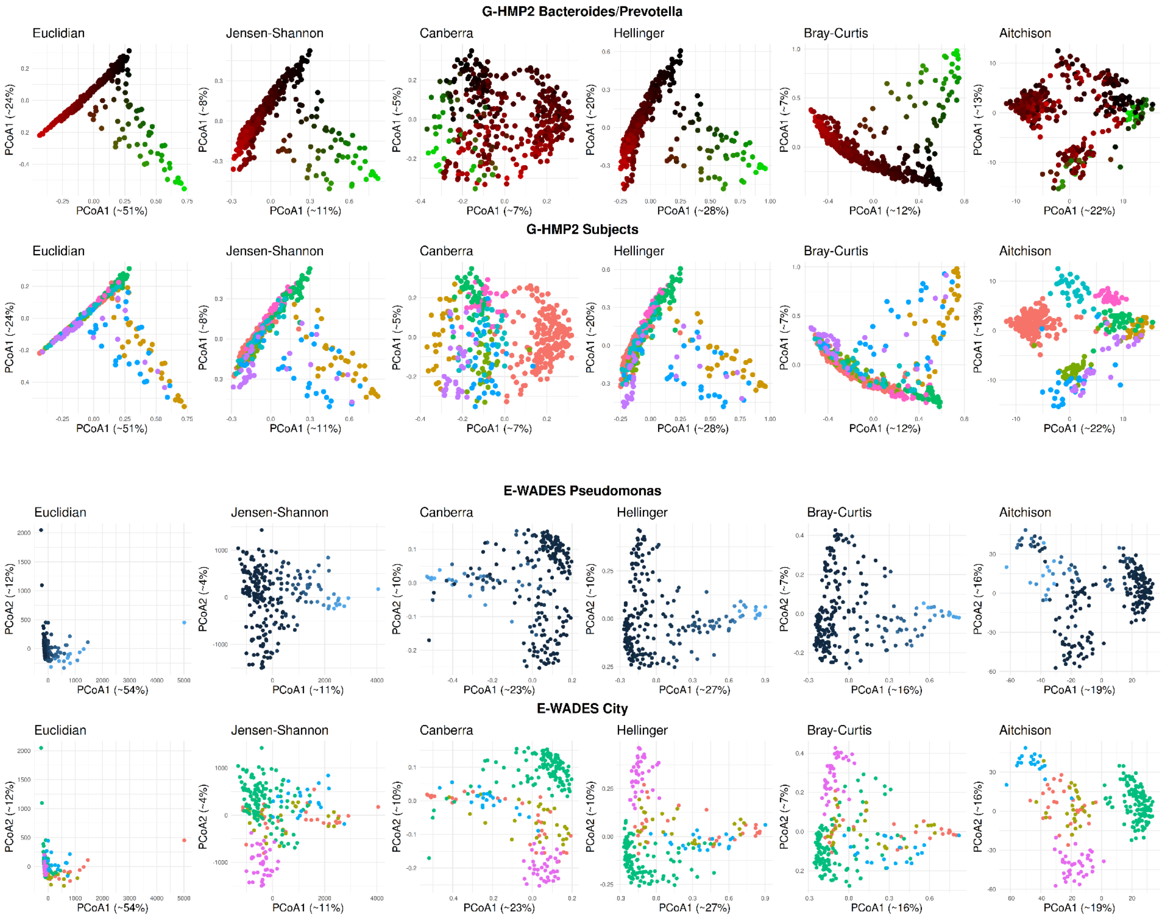

Supplementary Figure 1 | Principal Coordinate Analysis (PCoA) across multiple distance metrics and biological factors in G-HMP2 and E-WADES datasets.
The figure is divided into two main panels: the upper section shows results for the G-HMP2 dataset, and the lower section for the E-WADES dataset. In each panel, 12 PCoA plots are displayed, corresponding to six distance metrics (from left to right in each panel): Euclidean, Jensen-Shannon, Canberra, Hellinger, Bray-Curtis, and Aitchison.

Each metric is visualized with two alternative colorings that highlight distinct biological factors of interest. For G-HMP2, the top row is colored by the balance between *Bacteroides* and *Prevotella*, and the bottom row by subject identity. For E-WADES, the top row is colored by *Pseudomonas* abundance, and the bottom row by sampling city.

This comprehensive comparison illustrates that most non-compositional metrics (Euclidean, Jensen-Shannon, Canberra, Hellinger, and Bray-Curtis) produce similar ordination patterns primarily driven by dominant taxa. In contrast, Aitchison distance reveals clearer separations aligned with sample-specific groupings (i.e. the human subjects in G-HMP2 and the cities in E-WADES), reflecting how a compositional data analysis approach extracts different features.
Notably, in the Aitchison PCoA for G-HMP2, samples dominated by *Prevotella* are split into two distinct clusters, each corresponding to a different subject—highlighting how Aitchison distance preserves subject-specific compositional signatures beyond dominant taxa. Similarly, in the E-WADES dataset, samples with high

Pseudomonas abundance are positioned near the center of the Aitchison ordination space, suggesting that this dominant taxon is not the main driver of between-group differences, and supporting the metric's ability to reflect deeper structural variation in microbial communities as a function of regional localization.

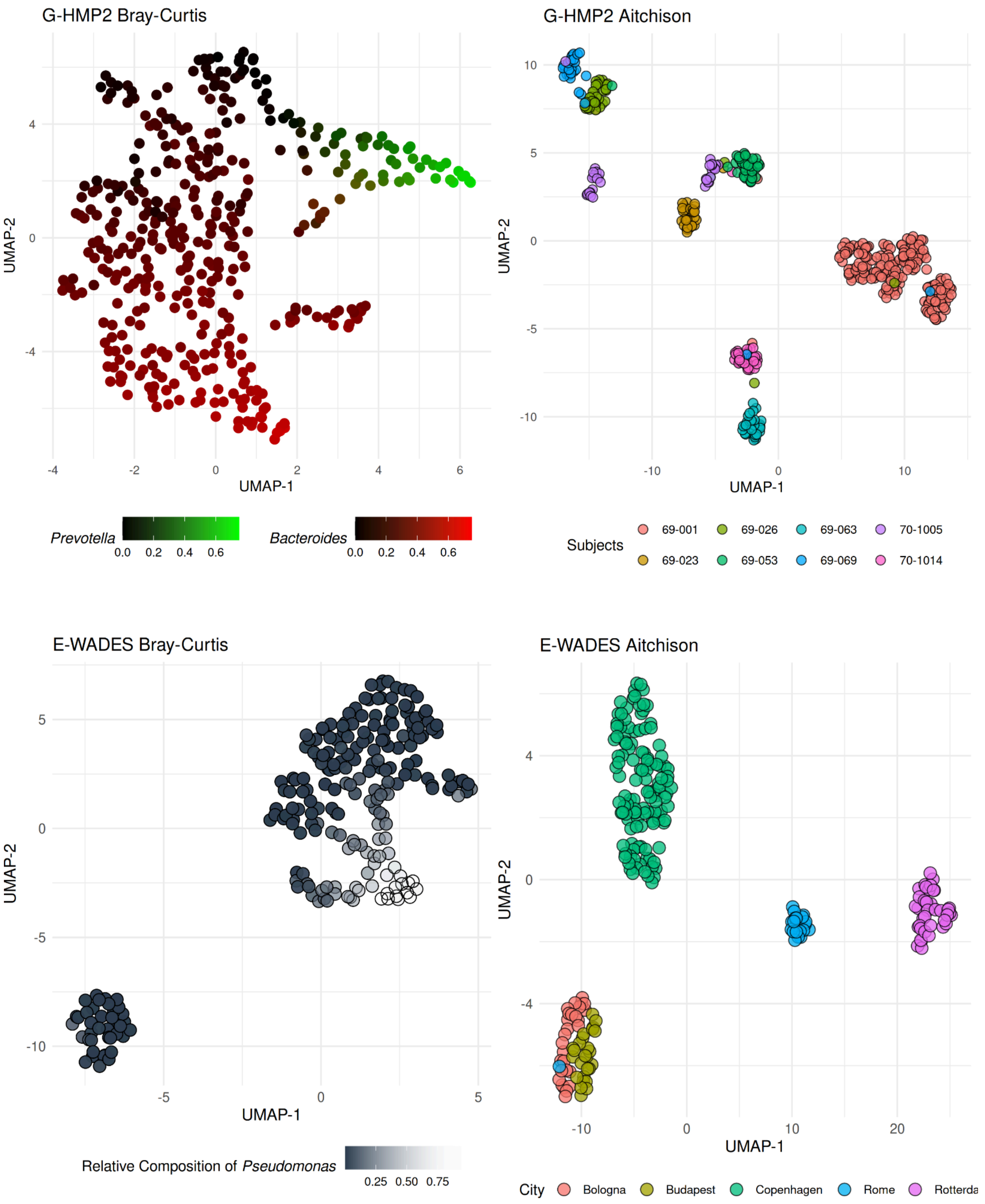

Supplementary Figure 2 | UMAP representation of microbial community structure using Bray-Curtis and Aitchison distances in G-HMP2 and E-WADES datasets.

This figure shows the comparisons as in Figures 1 and 2 of the main text (G-HMP2 and E-WADES datasets represented using Bray-Curtis and Aitchison metrics) but using UMAP (Uniform Manifold Approximation and Projection) instead of PCoA for dimensionality reduction. UMAP was computed with default parameters except for `min_dist = 0.5`, which controls how tightly points are allowed to cluster together in the low-dimensional space. As a non-linear method, UMAP typically better captures complex relationships in the data and reveals more distinct groupings compared to linear techniques.

In the G-HMP2 dataset, samples are colored by the relative abundance balance between *Bacteroides* and *Prevotella* (Bray-Curtis, left) or by subject identity (Aitchison, right). In the E-WADES dataset, samples are colored by the relative abundance of *Pseudomonas* (Bray-Curtis, left) or by sampling city (Aitchison, right).
The UMAP projections based on Aitchison distance show a clearer separation of samples belonging to the same individual—namely subjects in G-HMP2 and cities in E-WADES—which could support downstream clustering or classification analyses, even though group identities are known in this context. Conversely, Bray-Curtis projections remain heavily influenced by dominant taxa, particularly *Pseudomonas*. In the E-WADES dataset, the Bray-Curtis UMAP separates only the Rotterdam samples as a distinct group (bottom-left cluster), while the rest of the samples follow a continuous gradient primarily driven by the relative abundance of *Pseudomonas*, limiting the metric’s ability to reflect deeper structural differences between cities.
